# Supplementary material for: Resolving the full spectrum of human genome variation using Linked-Reads
Source: Genome Res. 2019 Apr;29(4):635–45. doi: 10.1101/gr.234443.118 (PMC6442396; doi:10.1101/gr.234443.118)
Supplement: Supplemental Material [file supp_gr.234443.118_Supplemental_longranger-2.2.zip › longranger-2.2.tar.bz2!/lr2.2-public/lib/stan/cmdstan/stan/doc/api/html/matrix__normal_8hpp_source.html]

Stan: src/stan/prob/distributions/multivariate/continuous/matrix\_normal.hpp Source File


|  |  |
| --- | --- |
|  | Stan  2.5.0  probability, sampling & optimization |


- Main Page
- Related Pages
- Namespaces
- Classes
- Files

- File List
- File Members

All Classes Namespaces Files Functions Variables Typedefs Enumerations Enumerator Friends Macros Pages

- src
- stan
- prob
- distributions
- multivariate
- continuous

matrix\_normal.hpp

Go to the documentation of this file.

1 #ifndef STAN\_\_PROB\_\_DISTRIBUTIONS\_\_MULTIVARIATE\_\_CONTINUOUS\_\_MATRIX\_NORMAL\_HPP

2 #define STAN\_\_PROB\_\_DISTRIBUTIONS\_\_MULTIVARIATE\_\_CONTINUOUS\_\_MATRIX\_NORMAL\_HPP

3

4 #include <stan/math/matrix\_error\_handling.hpp>

5 #include <stan/math/error\_handling.hpp>

6 #include <stan/prob/constants.hpp>

7 #include <stan/prob/traits.hpp>

8 #include <stan/agrad/rev.hpp>

9 #include <stan/meta/traits.hpp>

10 #include <stan/agrad/rev/matrix.hpp>

11 #include <stan/math/matrix/log.hpp>

12 #include <stan/math/matrix/log\_determinant.hpp>

13 #include <stan/math/matrix/subtract.hpp>

14 #include <stan/math/matrix/trace\_quad\_form.hpp>

15 #include <stan/math/error\_handling/matrix/check\_ldlt\_factor.hpp>

16 #include <stan/math/matrix/log\_determinant\_ldlt.hpp>

17

18 namespace stan {

19  namespace prob {

38  template <bool propto,

39  typename T\_y, typename T\_Mu, typename T\_Sigma, typename T\_D>

40  typename boost::math::tools::promote\_args<T\_y,T\_Mu,T\_Sigma,T\_D>::type

41  matrix\_normal\_prec\_log(const Eigen::Matrix<T\_y,Eigen::Dynamic,Eigen::Dynamic>& y,

42  const Eigen::Matrix<T\_Mu,Eigen::Dynamic,Eigen::Dynamic>& Mu,

43  const Eigen::Matrix<T\_Sigma,Eigen::Dynamic,Eigen::Dynamic>& Sigma,

44  const Eigen::Matrix<T\_D,Eigen::Dynamic,Eigen::Dynamic>& D) {

45  static const char\* function = "stan::prob::matrix\_normal\_prec\_log(%1%)";

46  typename boost::math::tools::promote\_args<T\_y,T\_Mu,T\_Sigma,T\_D>::type lp(0.0);

47

48  using stan::math::check\_not\_nan;

49  using stan::math::check\_symmetric;

50  using stan::math::check\_size\_match;

51  using stan::math::check\_positive;

52  using stan::math::check\_finite;

53  using stan::math::trace\_gen\_quad\_form;

54  using stan::math::log\_determinant\_ldlt;

55  using stan::math::subtract;

56  using stan::math::LDLT\_factor;

57  using stan::math::check\_ldlt\_factor;

58

59  check\_size\_match(function,

60  Sigma.rows(), "Rows of Sigma",

61  Sigma.cols(), "columns of Sigma",

62  &lp);

63  check\_positive(function, Sigma.rows(), "Sigma rows", &lp);

64  check\_finite(function, Sigma, "Sigma", &lp);

65  check\_symmetric(function, Sigma, "Sigma", &lp);

66

67  LDLT\_factor<T\_Sigma,Eigen::Dynamic,Eigen::Dynamic> ldlt\_Sigma(Sigma);

68  check\_ldlt\_factor(function,ldlt\_Sigma,"LDLT\_Factor of Sigma",&lp);

69  check\_size\_match(function,

70  D.rows(), "Rows of D",

71  D.cols(), "Columns of D",

72  &lp);

73  check\_positive(function, D.rows(), "D rows", &lp);

74  check\_finite(function, D, "D", &lp);

75  check\_symmetric(function, D, "Sigma", &lp);

76

77  LDLT\_factor<T\_D,Eigen::Dynamic,Eigen::Dynamic> ldlt\_D(D);

78  check\_ldlt\_factor(function,ldlt\_D,"LDLT\_Factor of D",&lp);

79  check\_size\_match(function,

80  y.rows(), "Rows of random variable",

81  Mu.rows(), "Rows of location parameter",

82  &lp);

83  check\_size\_match(function,

84  y.cols(), "Columns of random variable",

85  Mu.cols(), "Columns of location parameter",

86  &lp);

87  check\_size\_match(function,

88  y.rows(), "Rows of random variable",

89  Sigma.rows(), "Rows of Sigma",

90  &lp);

91  check\_size\_match(function,

92  y.cols(), "Columns of random variable",

93  D.rows(), "Rows of D",

94  &lp);

95  check\_finite(function, Mu, "Location parameter", &lp);

96  check\_finite(function, y, "Random variable", &lp);

97

98  if (include\_summand<propto>::value)

99  lp += NEG\_LOG\_SQRT\_TWO\_PI \* y.cols() \* y.rows();

100

101  if (include\_summand<propto,T\_Sigma>::value) {

102  lp += log\_determinant\_ldlt(ldlt\_Sigma) \* (0.5 \* y.rows());

103  }

104

105  if (include\_summand<propto,T\_D>::value) {

106  lp += log\_determinant\_ldlt(ldlt\_D) \* (0.5 \* y.cols());

107  }

108

109  if (include\_summand<propto,T\_y,T\_Mu,T\_Sigma,T\_D>::value) {

110  lp -= 0.5 \* trace\_gen\_quad\_form(D,Sigma,subtract(y,Mu));

111  }

112  return lp;

113  }

114

115  template <typename T\_y, typename T\_Mu, typename T\_Sigma, typename T\_D>

116  typename boost::math::tools::promote\_args<T\_y,T\_Mu,T\_Sigma,T\_D>::type

117  matrix\_normal\_prec\_log(const Eigen::Matrix<T\_y,Eigen::Dynamic,Eigen::Dynamic>& y,

118  const Eigen::Matrix<T\_Mu,Eigen::Dynamic,Eigen::Dynamic>& Mu,

119  const Eigen::Matrix<T\_Sigma,Eigen::Dynamic,Eigen::Dynamic>& Sigma,

120  const Eigen::Matrix<T\_D,Eigen::Dynamic,Eigen::Dynamic>& D) {

121  return matrix\_normal\_prec\_log<false>(y,Mu,Sigma,D);

122  }

123  }

124 }

125

126 #endif

matrix.hpp

traits.hpp

stan::math::check\_size\_match

bool check\_size\_match(const char \*function, T\_size1 i, const char \*name\_i, T\_size2 j, const char \*name\_j, T\_result \*result)

**Definition:** check\_size\_match.hpp:13

error\_handling.hpp

trace\_quad\_form.hpp

stan::math::check\_finite

bool check\_finite(const char \*function, const T\_y &y, const char \*name, T\_result \*result)

Checks if the variable y is finite.

**Definition:** check\_finite.hpp:52

stan::agrad::trace\_gen\_quad\_form

fvar< T > trace\_gen\_quad\_form(const Eigen::Matrix< fvar< T >, RD, CD > &D, const Eigen::Matrix< fvar< T >, RA, CA > &A, const Eigen::Matrix< fvar< T >, RB, CB > &B)

**Definition:** trace\_gen\_quad\_form.hpp:15

log.hpp

stan::prob::matrix\_normal\_prec\_log

boost::math::tools::promote\_args< T\_y, T\_Mu, T\_Sigma, T\_D >::type matrix\_normal\_prec\_log(const Eigen::Matrix< T\_y, Eigen::Dynamic, Eigen::Dynamic > &y, const Eigen::Matrix< T\_Mu, Eigen::Dynamic, Eigen::Dynamic > &Mu, const Eigen::Matrix< T\_Sigma, Eigen::Dynamic, Eigen::Dynamic > &Sigma, const Eigen::Matrix< T\_D, Eigen::Dynamic, Eigen::Dynamic > &D)

The log of the matrix normal density for the given y, mu, Sigma and D where Sigma and D are given as ...

**Definition:** matrix\_normal.hpp:41

matrix\_error\_handling.hpp

constants.hpp

traits.hpp

stan::math::check\_symmetric

bool check\_symmetric(const char \*function, const Eigen::Matrix< T\_y, Eigen::Dynamic, Eigen::Dynamic > &y, const char \*name, T\_result \*result)

Return true if the specified matrix is symmetric.

**Definition:** check\_symmetric.hpp:31

stan::math::check\_ldlt\_factor

bool check\_ldlt\_factor(const char \*function, stan::math::LDLT\_factor< T, R, C > &A, const char \*name, T\_result \*result)

Return true if the underlying matrix is positive definite.

**Definition:** check\_ldlt\_factor.hpp:24

check\_ldlt\_factor.hpp

stan::prob::include\_summand

Template metaprogram to calculate whether a summand needs to be included in a proportional (log) prob...

**Definition:** traits.hpp:35

stan::math::subtract

Eigen::Matrix< typename boost::math::tools::promote\_args< T1, T2 >::type, R, C > subtract(const Eigen::Matrix< T1, R, C > &m1, const Eigen::Matrix< T2, R, C > &m2)

Return the result of subtracting the second specified matrix from the first specified matrix...

**Definition:** subtract.hpp:27

rev.hpp

stan::math::LDLT\_factor

**Definition:** LDLT\_factor.hpp:16

log\_determinant\_ldlt.hpp

log\_determinant.hpp

stan::math::check\_positive

bool check\_positive(const char \*function, const T\_y &y, const char \*name, T\_result \*result)

**Definition:** check\_positive.hpp:54

stan::math::check\_not\_nan

bool check\_not\_nan(const char \*function, const T\_y &y, const char \*name, T\_result \*result)

Checks if the variable y is nan.

**Definition:** check\_not\_nan.hpp:56

stan::agrad::log\_determinant\_ldlt

var log\_determinant\_ldlt(stan::math::LDLT\_factor< var, R, C > &A)

**Definition:** log\_determinant\_ldlt.hpp:48

stan::math::log\_determinant\_ldlt

T log\_determinant\_ldlt(stan::math::LDLT\_factor< T, R, C > &A)

**Definition:** log\_determinant\_ldlt.hpp:12

stan::math::trace\_gen\_quad\_form

double trace\_gen\_quad\_form(const Eigen::Matrix< double, RD, CD > &D, const Eigen::Matrix< double, RA, CA > &A, const Eigen::Matrix< double, RB, CB > &B)

Compute trace(D B^T A B).

**Definition:** trace\_gen\_quad\_form.hpp:17

subtract.hpp


---

[ Stan Home Page ]

*© 2011–2014,
Stan Development Team.*
